# Acute reversible rhabdomyolysis during direct-acting antiviral hepatitis C virus treatment: a case report

**DOI:** 10.1186/s13256-021-03138-0

**Published:** 2021-12-19

**Authors:** Abdulrahman Qatomah, Majidah Bukhari, Edward Cupler, Hosam Alardati, Mohammad Mawardi

**Affiliations:** 1 Department of Internal Medicine, Royal Victoria Hospital, McGill University, 1001 Decarie Blv , Montréal , H4A 3J1 Canada; 2 Department of Neurology, King Faisal Specialist Hospital and Research Center, Prince Sultan Street, Ar Rawdah, Jeddah, 23433 Saudi Arabia; 3 Department of Histopathology, King Faisal Specialist Hospital and Research Center, Prince Sultan Street, Ar Rawdah, Jeddah, 23433 Saudi Arabia

**Keywords:** Direct antiviral therapy, Rhabdomyolysis, Creatine kinase, Sofosbuvir, Case report

## Abstract

**Introduction:**

Treatment of hepatitis C infection has evolved dramatically since 2011. Previous conventional therapy with interferon and ribavirin used to have a low sustained virological response rate of less than 40%. In the new direct-acting antiviral therapy era, a sustained virological response can be achieved in more than 90% of cases.

**Case presentation:**

We report a rare case of severe reversible acute rhabdomyolysis in a 31-year-old Saudi male patient with very long-chain acyl-coenzyme A dehydrogenase deficiency and chronic hepatitis C infection. The patient was clinically asymptomatic with no signs of decompensated liver disease.

The patient received new direct-acting antiviral agents: sofosbuvir and daclatasvir. Fourteen days after initiation of direct-acting antiviral agents, the patient was found to have asymptomatic rhabdomyolysis. His creatine kinase peaked at 2572 IU/l, and he was treated conservatively; the direct-acting antiviral agents were discontinued and within 7 days, the patient’s creatine kinase levels normalized.

**Conclusion:**

This case highlights possible direct-acting antiviral agent-induced rhabdomyolysis in a patient with very-long-chain acyl-CoA dehydrogenase deficiency, presumably through alteration of mitochondrial membrane potential. Further studies are required to assess the possible impact and associations.

## Background

Hepatitis C viral infection is one of the most common causes of chronic liver disease worldwide [1]. Therapy of hepatitis C infection (HCV) has dramatically improved over the past few decades with the introduction of direct-acting antiviral agents (DAAs). In the era of new direct-acting antiviral therapy, a sustained virologic response can be achieved in more than 90% [2] of cases, with fewer adverse events and higher tolerability than previous conventional therapy with interferon-based regimens.

Rhabdomyolysis is a syndrome caused by injury to skeletal muscles involving the leakage of a high volume of toxic substances into plasma, including myoglobin with lysis of skeletal muscle cells. Rhabdomyolysis is an adverse event that occurs with DAA use, mainly due to drug–drug interactions with concomitant use of HMG-CoA reductase inhibitors (statins) and other myotoxic agents [3]. Rhabdomyolysis can develop in patients diagnosed with very long-chain acyl-coenzyme A dehydrogenase (VLCAD) deficiency. VLCAD disease is an autosomal recessive [4] mitochondrial fatty acid oxidation disorder that is associated with recurrent rhabdomyolysis, with an estimated prevalence of 1 in 42,500 [5–7]. It represents one of the beta-oxidation defects that can involve one of the essential enzymatic components for fatty acid metabolism. VLCAD is a key enzyme catalyzing the first reaction in the mitochondrial beta-oxidation of long-chain fatty acids with a length of 14–20 carbons. VLCAD can present either with severe early-onset disease, an intermediate severity during childhood, or as a mild adult-onset disease [8]. l-carintine is considered a therapeutic option that minimizes the chance of recurrent rhabdomyolysis. Affected patients are typically placed on a low-fat and high-carbohydrate diet.

## Case presentation

We report a 31-year-old Saudi male patient who presented initially to the neurology clinic at King Faisal Specialist Hospital and Research Centre, Jeddah, to evaluate reversible recurrent rhabdomyolysis. The patient was healthy and had no medical background prior to this presentation. He denied the use of any medications or herbal agents. His family history was negative for any neurological illnesses, and his parents were not related. The patient was unemployed at the time of the first presentation. He habitually smoked one pack of cigarettes per day for 10 years, however, he had no history of alcohol intake or illicit drug use.

## Clinical findings

The initial clinical examination at the neurology clinic, including vital signs and neurological examination, was entirely within normal range, with a calculated body mass index (BMI) of 30. Further physical examination at the hepatology clinic identified no signs of decompensated liver disease.

## Timeline

$${\mathrm{Initiation}}\;{\mathrm{of}}\;{\mathrm{treatment}}\mathop{\longrightarrow}\limits^{{14\;{\mathrm{days}}}}{\mathrm{CK}}\;{2572}\;{{{\mathrm{IU}}} \mathord{\left/ {\vphantom {{{\mathrm{IU}}} {\mathrm{L}}}} \right. \kern-\nulldelimiterspace} {\mathrm{L}}},{\mathrm{DAA}}\;{\mathrm{discontinued}}\mathop{\longrightarrow}\limits^{{7\;{\mathrm{days}}}}{\mathrm{Normal}}\;{\mathrm{CK}}$$

## Diagnostic assessment

The patient’s initial workup showed normal results for nerve conduction and electromyography. Muscle biopsy showed findings consistent with myopathy type 1 muscle fiber atrophy. Further testing with whole-exome sequencing confirmed a homozygous variant of acyl-CoA dehydrogenase very long chain (ACADVL), a variant of unknown significance, consistent with the diagnosis of very long-chain acyl-CoA dehydrogenase deficiency (VLCAD) disease. The patient was started on l-carnitine supplements. Further follow-up of the patient showed less frequent episodes of rhabdomyolysis.

Hepatitis c virus was positive during the workup process, at which point the patient was referred to the hepatology clinic where a thorough assessment was conducted. The patient was on l-carnitine supplements at the time of referral and denied a history suggestive of decompensation of liver disease. He continues to be under regular follow-up by the neurology service with a stable clinical status. Regarding risk factors of hepatitis C, he denied any history of drug abuse, had no history of blood transfusion, and no family history of liver disease.

Patient investigations showed HCV, polymerase chain reaction (PCR), ribonucleic acid (RNA) 182624 IU/ml, with genotype 4, alanine aminotransferase (ALT) 51 μ/l, aspartate aminotransferase (AST) 30 μ/l, alkaline phosphatase 85 U/l, total bilirubin 9 μmol/l, prothrombin time (PT) 11.2 seconds, partial thromboplastin time (PTT) 30.8 seconds, international normalized ratio (INR) 1.0, creatine kinase 546 U/l, creatinine 73 mmol/l, hemoglobin 150 g/l, white blood count 6900 × 10^9^, and platelet count 309,000 × 10^9^.

**Fig. 1:**
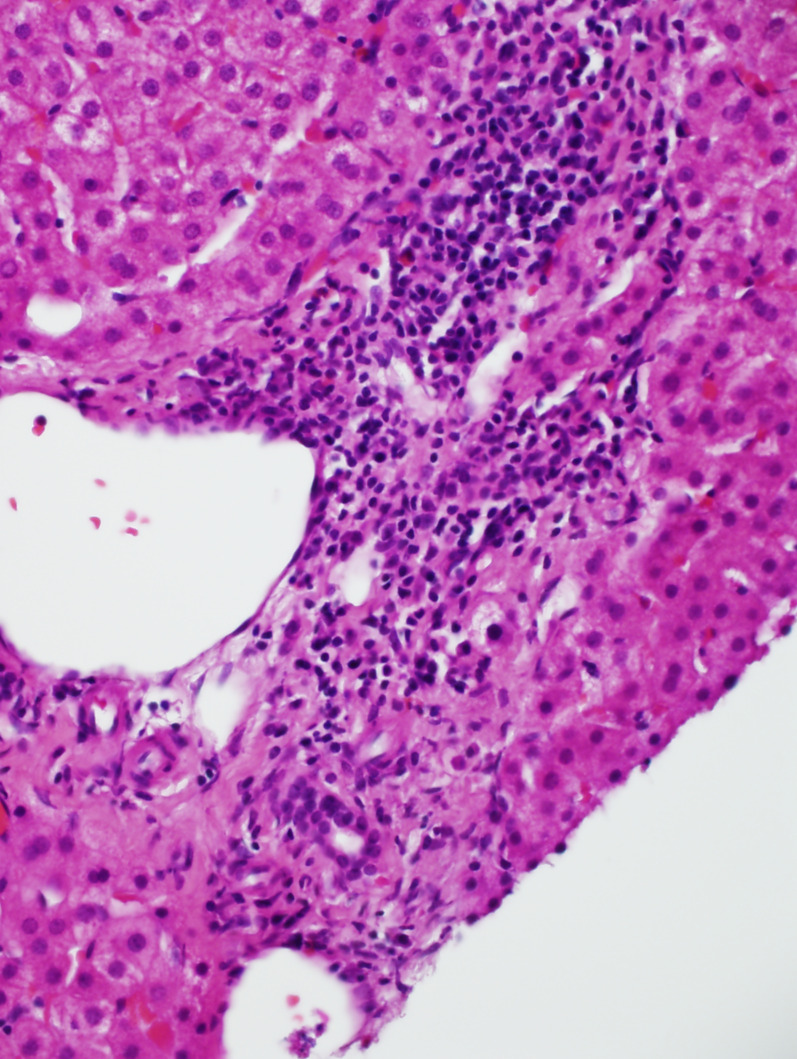
The core biopsy of the liver showed preserved architecture with patchy mild lymphocytic infiltrates in the portal tract with rare eosinophils. No changes to bile ducts or portal vessels. Rare hepatocytic cell necrosis present. Minimal steatosis. No bridging fibrosis.

Abdominal ultrasound was unremarkable with normal liver parenchyma and no signs of portal hypertension. Liver biopsy showed periportal inflammation consistent with chronic HCV infection grade 1 stage 0 fibrosis (Fig. 1).

## Therapeutic intervention

The patient was initially treated with an interferon-based regimen, which was discontinued due to the development of rhabdomyolysis. A decision was made to treat conservatively as direct-acting antivirals were not available. Three years later, after DAA became available, he opted to start on DAA after counseling. He was started on a combination of sofosbuvir 400 mg orally once daily and daclatasvir 60 mg orally once daily, with a plan to complete a treatment course of 12 weeks.

## Follow-up and outcome

Fourteen days following initiation of DAA therapy, the patient’s repeated blood work showed CK levels at 2572 IU/l. He was asymptomatic at the time of follow-up. After ruling out all contributing factors, he was treated conservatively with discontinuation of DAA. Seven days after discontinuation of DAA, the repeated CK levels were back to normal range (< 198 U/l). A follow-up HCV PCR showed a viral load of 863924 IU/ml. A decision was made to continue with the discontinuation of DAA and monitor clinically and follow HCV PCR. Six months after discontinuation, the patient was seen and assessed; he continued to be asymptomatic and follow-up HCV PCR showed a viral load of 1811560 IU/ml, alpha-fetoprotein 4 mcg/l, ALT 59 U/l, AST 30 μ/l, ALP 139 μ/l, total bilirubin 7 mmol/l, and an updated Fibroscan of F0.

## Discussion

In summary, a 31-year-old male patient presented to our institution with a history of recurrent rhabdomyolysis; further investigation confirmed the diagnosis of VLCAD deficiency and concomitant chronic HCV infection. The patient had recurrent rhabdomyolysis induced by DAA treatment.

For a patient with VLCAD and ACADL gene mutation, there is an impairment in catalyzing the first step of mitochondrial β-oxidation [9]. The disease manifests as rhabdomyolysis, which occurs with stressors such as physical exercise [10] and dehydration. DAA for hepatitis C virus (HCV) infection has shown a significant sustained response with fewer tolerable adverse reactions.

Rhabdomyolysis as an adverse reaction to DAA alone has not been observed nor documented in the literature. However, an interaction that resulted from a combination of DAA with 3-hydroxy-3-methyl-glutaryl-coenzyme A Reductase (HMG-COA) inhibitors (statins) reductase inhibitor and colchicine was reported. The mechanism is presumed to be via coadministration of sofosbuvir/ledipasvir with rosuvastatin, in which ledipasvir will inhibit the drug-transported breast cancer resistance protein (BCRP) leading to a marked increase in plasma concentration of rosuvastatin.

Sofosbuvir is a nucleos(t)ide analog (NA) that works by inhibiting non-structural protein 5B (NS5B). NA has shown an affinity towards mitochondrial polymerases, hence mitochondrial damage and cell death [8].

The exact mechanism by which DAA induced rhabdomyolysis in a patient with VLCAD is not well understood.

However, DAA may affect unhealthy mitochondria, which eventually leads to cellular damage manifesting as rhabdomyolysis, as demonstrated in our case.

## Conclusion

This case highlights possible DAA-induced rhabdomyolysis in a patient with very long-chain acyl-CoA dehydrogenase deficiency. The precise mechanism is not well understood; however, it is thought to be via altering mitochondrial membrane potential, subsequently inducing cellular damage. We believe that rhabdomyolysis should be considered when treating HCV with DAA in any patient myopathies.

To our knowledge, this is the first reported case in the literature. Further studies are needed to assess the possible impact and association of such interactions.
